# The Armor of the Chinese Sturgeon: A Study of the Microstructure and Mechanical Properties of the Ventral Bony Plates

**DOI:** 10.3390/mi14020256

**Published:** 2023-01-19

**Authors:** Yu Zheng, Xin Li, Ping Liu, Ying Chen, Ce Guo

**Affiliations:** 1 College of Mechanical and Electrical Engineering, Suqian University, Suqian 223800, China; 2 Institute of Bio-Inspired Structure and Surface Engineering, College of Mechanical and Electrical Engineering, Nanjing University of Aeronautics and Astronautics, Nanjing 210016, China

**Keywords:** ventral bony plates, morphology, mechanical properties, protective structures

## Abstract

Benefiting from their unique morphological characteristics and structural properties, the ventral bony plates of the Chinese sturgeon are excellent biological protective tissue. In this work, we studied the micro- and macro-morphology and mechanical properties of the ventral bony plates of the Chinese sturgeon to elucidate the special protective mechanisms of the bony plates. Experiments involving scanning electron microscopy and energy-dispersive X-ray spectroscopy revealed that the bony plates possess a hierarchical structure and a ridge-like shape. This structure comprises a surface layer containing mineralized nanocrystals and an internal layer containing mineralized collagen fibers. From the surface layer to the internal layer, the degree of mineralization decreases gradually. Nanoindentation, tension, and compression tests demonstrated that the bony plates feature excellent mechanical properties and a high specific tensile strength comparable to that of stainless steel. Moreover, water can significantly improve the fracture toughness and deformability of the bony plates and effectively enhance the damage tolerance of the structures. The obtained results concerning the microstructure–property–function relationships of the ventral bony plates of the Chinese sturgeon may provide novel insights for designing protective structures that are both lightweight and high strength.

## 1. Introduction

Over hundreds of millions of years of evolution, as the first protective barrier in the fish body, fish scales have evolved into biological structural materials with low weight, high strength, and remarkable toughness. Moreover, the overlapping scale structure not only provides good flexibility but also disperses loads such as those occurring during bites and impact, thus improving the deformation and protection performance [1]. Owing to differences in the living environment, the structural characteristics and biomechanical properties of fish scales vary considerably among different species [2,3,4,5].

According to previous research, fish scales can be divided into cycloid scales, ctenoid scales, ganoid scales, placoid scales, and bony plates [5,6]. The scales of most fish species are either cycloid scales or ctenoid scales, as in the case of *Arapaima gigas*, *Morone saxatilis*, *Carassius auratus*, *Cyprinus carpio*, and *Paramisgurnus dabryanus* [7,8,9,10,11]. These two scale types are both composed of a bony layer and a collagen layer and exhibit good deformability. The collagen layer has a low mineralization degree, and the helically stacked fibers can effectively inhibit crack propagation and prevent fracture failure of the scale [7,8].

Ganoid scales are found in a few fish species, such as *Atractosteus spatula* and *Polypterus senegalus* [12,13]. These scales are composed of a ganoid layer and a bony layer, and the main component of the two layers is hydroxyapatite. Compared with the bony layer, the ganoid layer displays a higher degree of mineralization with a hardness of approximately 2.5 GPa [12]. Ganoid scales possess higher strength and hardness compared with cycloid scales and ctenoid scales but exhibit inferior deformability.

Placoid scales occur on the body surface of sharks and are a typical drag-reducing structure [14]. The ridge structure and arc groove on the scale surface effectively improve the fluid dynamics and flow state around the fish body and reduce the shear pressure, transverse vortex formation, and fluid separation during swimming [15].

In contrast to the other scale types, bony plates contain only the bony layer. Song et al. studied the bony plates of *Gasterosteus aculeatus,* and the results revealed that the internal layer of the bony plates comprises a porous sandwich structure, which endows the bony plates with greater protective performance against penetrating loads than a densely configured structure [16]. However, the mechanical properties of the bony plates, such as their tensile strength and deformation characteristics, have not been studied.

Inspired by the outstanding protective properties of fish scales, numerous researchers have designed flexible protective structures [11,17,18,19]. These structures are composed of a bionic scale layer and a flexible substrate layer, where the bionic scale layer is used to resist impact loads and the flexible substrate layer absorbs the impact energy through large deformation. These biomimetic structures all require a flexible and thick substrate material for absorbing the impact energy via deformation, thus leading to bulky and heavy protective structures. In this paper, we introduce a unique fish scale structure and hope to provide a new design concept for protective structures that are both lightweight and high strength.

The Chinese sturgeon (*Acipenser sinensis*) is regarded as a living fossil and is a benthic fish with a fusiform shape [20,21]. Unlike most fish species, most of a sturgeon’s body surface is covered solely by fish skin, and the bony plates only cover a few body areas. The Chinese sturgeon is a migratory species with a migration distance exceeding 3000 km [22,23]. During this long journey, the juvenile second filial generation of Chinese sturgeons may suffer many dangerous events, such as being attacked by predators or colliding with boats and ships when they are accidentally caught by fishermen [23,24]. Therefore, the bony plates covering parts of the sturgeon’s body surface probably play a key protective role. Moreover, most of the scales are oval or quadrangular, whereas the bony plates on the ventral side of the body display a special configuration. The defensive characteristics of this distinct structure and the cooperative protective mechanism between bony plates have not been studied. Previous studies on the Chinese sturgeon have primarily focused on its biological evolution, sex identification, and eco-environmental protection [25,26,27]. In this work, we present a detailed analysis of the structural morphology and protective mechanisms of the ventral bony plates of the Chinese sturgeon to experimentally assess their mechanical contribution to the defensive function and hope to provide motivation for designing bioinspired lightweight and high-strength protective structures.

## 2. Materials and Methods

### 2.1. Materials and Experiments

Six Chinese sturgeons (captive bred) for experimental testing were acquired from a local fish store (Nanjing, Jiangsu Province). The ventral bony plates of the sturgeons were dissected from the fish body using a scalpel, tweezers, and surgical scissors, and the skin tissue on the plate surface was brushed off with a soft bristle brush under a biological microscope. Next, the bony plates were immersed in freshwater and subjected to ultrasonic cleaning for 20 min. The prepared samples were dried at room temperature (ca. 25 °C) for 6 h then sealed in labeled plastic bags, which were stored in a refrigerator (5 °C) prior to the morphological observations and mechanical tests. Scanning electron microscopy (SEM) and energy-dispersive X-ray (EDX) spectroscopy were used to examine the microstructure and composition of the ventral bony plates. Moreover, nanoindentation, tension, and compression tests were performed on the bony plates to further evaluate their mechanical response. The obtained results were expected to help us better understand the microstructure–property–function relationships of this material. The SEM, EDX, and nanoindentation experiments were performed at the Nanjing Institute of Mechanical and Electrical Hydraulic Engineering. The mechanical properties of the bony plates were investigated at the Nanjing Institute of Technology.

### 2.2. SEM and EDX Analysis

The bony plates were removed from the sealed bags, soaked in fresh water for 1 h to bring them to room temperature (25 °C), and then immersed in aqueous ethanol solutions of various concentrations (50%, 70%, 90%, and 100%) for dehydration, where the dehydration time in each solution was 20 min. The samples were then dried at room temperature, and specimens were prepared for observation using a scalpel and medical scissors. Finally, the prepared specimens were fixed on a cylindrical table with conductive adhesive and then analyzed by SEM (FEI Quanta 650, Portland, OR, USA) and EDX spectroscopy (Oxford AZtecLive and UltimMax, Concord, MA, USA).

### 2.3. Nanoindentation Experiments

Nanoindentation samples with dimensions of 4 mm × 2 mm were prepared using a scalpel and tweezers. Prior to the tests, the sample surfaces were ground with abrasive paper (0.3 and 0.05 μm) to obtain a smooth surface for greater accuracy. Because the specimens were small and thin, the grinding process was performed slowly to avoid damaging the internal structures of the bony plates. The prepared samples were immersed in freshwater and subjected to ultrasonic cleaning for 20 min to remove surface debris. Next, the samples (*n* = 6) were divided into two groups: the first group (*n* = 3) was soaked in fresh water for 3 d (the water was replaced every 12 h) to prepare wet samples, whereas the second group (*n* = 3) was dried at room temperature (ca. 25 °C) for one week to prepare dry samples. Finally, an in-situ nanoindentation instrument (FemtoTools FT-NMT04, Buchs, Switzerland) was used to determine the elastic modulus and hardness of the prepared samples.

### 2.4. Tension/Compression Tests

**Figure 1:**
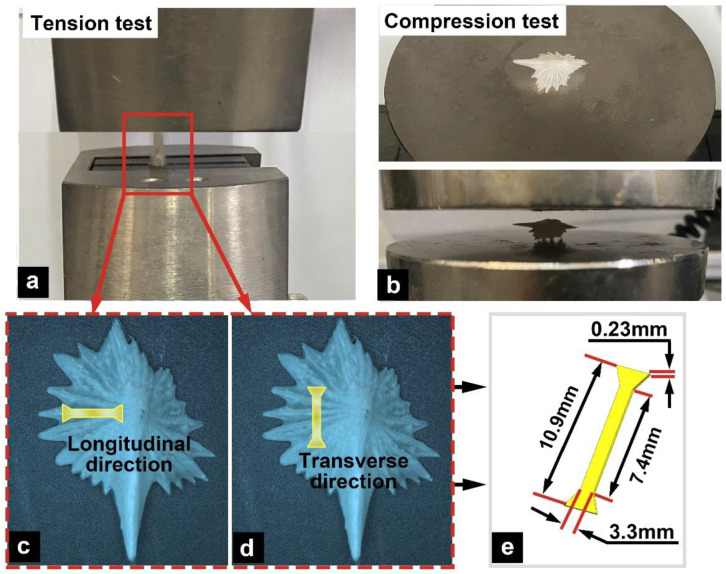
Tension and compression tests. (**a**) Tension tests in two directions: (**c**) the longitudinal direction and (**d**) the transverse direction, where the tension sample (**e**) possessed a dumbbell shape with a size of 7.4 × 3.3 × 0.23 mm^3^. (**b**) Compression test.

To further analyze the mechanical properties of the ventral bony plates of the Chinese sturgeon, tension tests were conducted in different directions (longitudinal and transverse directions; Figure 1a,c,d) and in different states (dry and wet). Prior to these tests, dumbbell-shaped tensile samples were prepared, and the specimen size was 7.4 × 3.3 × 0.23 mm^3^ (Figure 1e). The number of samples for each direction and state was *n* = 3. The methods for preparing the dry and wet samples were the same as those described in Section 2.3. The tension tests were performed on an electronic universal testing machine (CMT 4503, Nanjing, China) at a speed of 1 mm/min.

The ventral bony plates exhibited a ridge-like shape. Compared with the common flat fish scales, this structural feature presumably endows the bony plates with greater deformability. To analyze the relationship between the hierarchical structure of the bony plates and their mechanical response and to evaluate the impact of water on the toughness of the bony plates, we conducted the tests for both dry (*n* = 6) and wet (*n* = 6) samples (Figure 1b). The tests were performed on an electronic universal testing machine (CMT 4503, China) at a compression speed of 0.5 mm/min.

## 3. Results

### 3.1. Arrangement Feature

**Figure 2:**
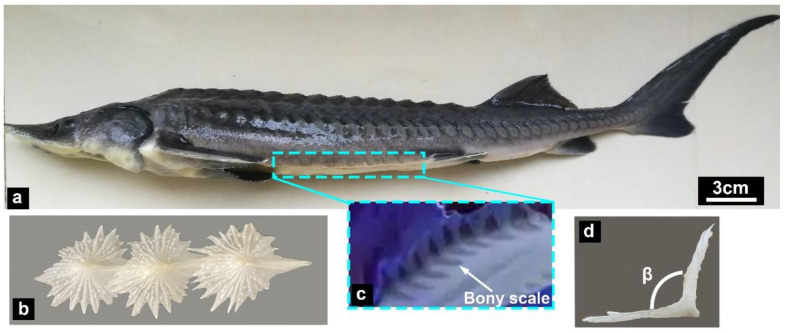
Ventral bony plates of the Chinese sturgeon (Acipenser sinensis). (**a**) Photograph of a Chinese sturgeon. (**b**) The ventral bony plates dissected from a Chinese sturgeon. (**c**) Arrangement characteristics of the ventral bony plates. (**d**) Ridge-like structure of the bony plates.

Figure 2 presents the morphological characteristics of the Chinese sturgeon and the structural morphology of its ventral bony plates. It should be noted that the ventral bony plates are covered by the fish skin epidermis [28]. The arrangement characteristics of the ventral bony plates were observed under ultraviolet light (Figure 2c), and the results revealed that the bony plates exhibited a ridge-like shape. Furthermore, the bony plates possessed an asymmetric structure with a long crest structure in the center (Figure 2b). This crest structure was thicker than the bony plates on either side. The angle β between the bony plates was ~105.5° (Figure 2d). As a benthic fish, the bony plates located on the ventral side could effectively protect the sturgeon’s body.

### 3.2. Microstructural Characterization

**Figure 3:**
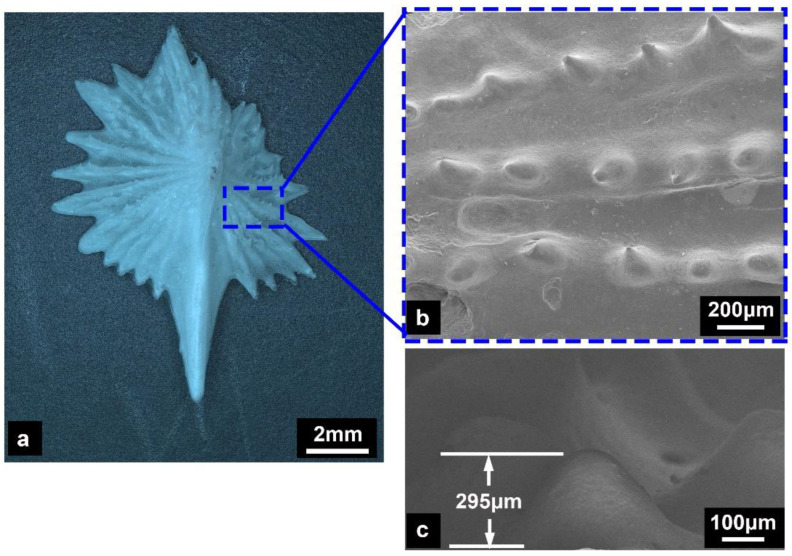
Structural characterization of the ventral bony plates. (**a**) The surface morphology. (**b**,**c**) Microstructure of the nodular ridges.

Figure 3 depicts the microscopic morphological characteristics of the ventral bony plate. The bony plate featured an asymmetric structure (Figure 3a), and its surface was covered with nodular ridges that extended from the crest to the serrated edges (Figure 3b). The height of the ridges was 200–300 μm (Figure 3c). Previous results have suggested that nodular ridges on the surface of fish scales are conducive to reducing swimming resistance [7,13,29]. However, the ventral bony plates of the Chinese sturgeon were completely covered by the fish skin epidermis, and there probably the presence of the epidermis directly connected to the bony elements. This result indicates that nodular ridges primarily enhance the connection between soft tissues of the skin and the bony elements.

**Figure 4:**
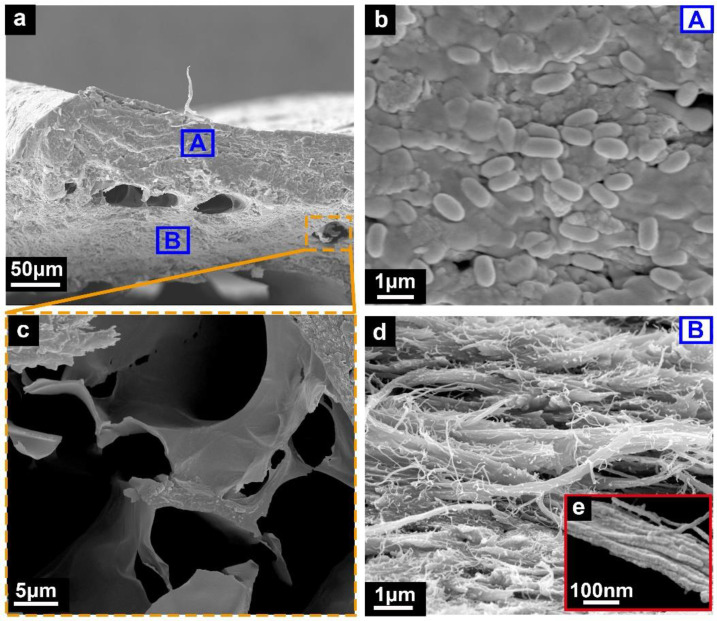
Cross-sections of the ventral bony plates. (**a**) Cross-section of the bony plates. (**b**–**d**) Details from the surface layer (A) to the internal layer(B). (**e**) Collagen fibrils with a diameter of ca. 10 nm.

The sectional microstructures of the ventral bony plates are shown in Figure 4. The bony plates, with a thickness of 200–300 μm, displayed a high degree of mineralization. The bony plates possessed a hierarchical structure (Figure 4a), in which the surface layer was composed of mineralized nanocrystals (Figure 4b). The dense structure formed by these tiny nanocrystals could significantly increase the strength and hardness of the surface layer. Meanwhile, the internal layer of the bony plates was composed of mineralized collagen fibers, where the diameter of the fibrils was approximately 10 nm (Figure 4d,e). The degree of mineralization of the fiber structures in the internal layer was similar to that of mineralized chitin [30], which is much higher than that of the collagen fibers in cycloid scales and ctenoid scales. In addition, the bony plates exhibited porous structural characteristics (Figure 4a), and residual collagen was found inside the pores (Figure 4c). During the ossification of the bony plates of the Chinese sturgeon, most of the collagen in the pores leaches out and undergoes mineralization [28]. Any residual collagen was observed in the pores as mineralized collagen. These pores could effectively reduce the weight of the bony plates and absorb energy via deformation.

### 3.3. Component Analysis

**Figure 5:**
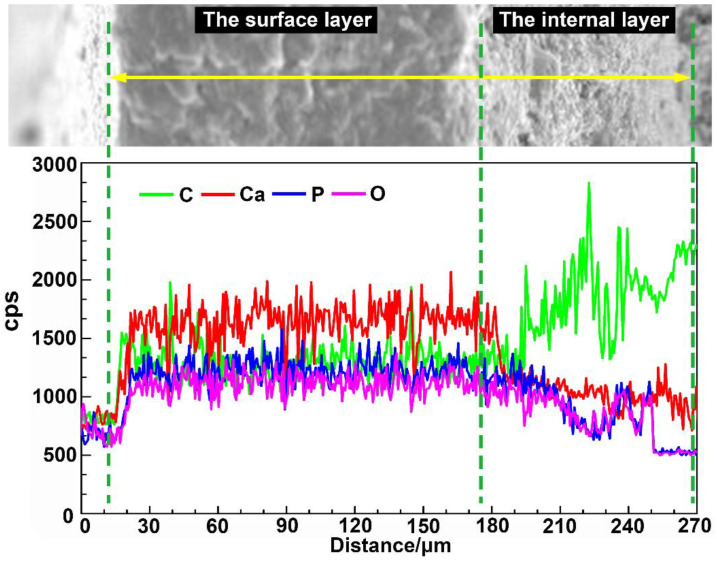
Composition analysis of the ventral bony plates with respect to the cross-sectional distance.

The elemental contents (C, Ca, P, and O) of the different structural layers are presented in Figure 5 and Table 1, where higher cps values correspond to a higher elemental content/concentration. The test results revealed that the main component of the bony plates was hydroxyapatite (HAP), which is widely found in animal bones and fish scales and can significantly increase the hardness and strength of biological structures. From the surface layer to the internal layer, the contents of Ca, P, and O in the bony plates gradually decreased, while the content of C increased. These variations indicate that the surface layer of the bony plates was highly mineralized, while the content of organic components, such as fiber structures, was higher in the internal layer. The results from the composition tests were in good agreement with the microscopic morphological analysis.

### 3.4. Nanoindentation Tests

**Figure 6:**
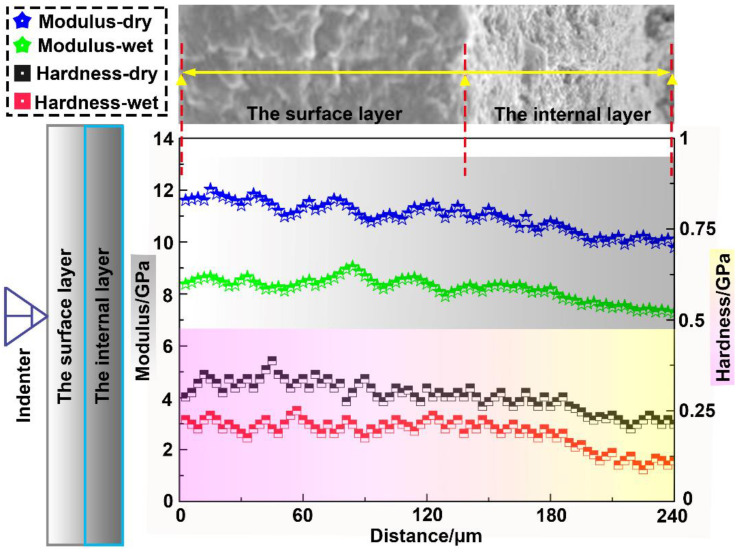
Elastic modulus and hardness curves for the ventral bony plates.

Figure 6 presents the characteristic elastic modulus and hardness curves for the dry and wet specimens as a function of the indentation depth. Table 2 summarizes the average elastic modulus and hardness values of each structural layer for the dry and wet samples. It can be seen that, with increasing indentation depth, the elastic modulus and hardness of the bony plates gradually decreased from the surface layer to the internal layer, which indicated that the mineralization degree of the internal layer was lower. The mechanical properties of biological structures are closely related to their degree of mineralization [13]: a high degree of mineralization can effectively increase the elastic modulus and hardness, thereby improving the deformation resistance.

Furthermore, it can be calculated from Table 2 that the average elastic modulus and hardness values were 11.37 ± 0.59 and 0.28 ± 0.04 GPa for the dry samples and 8.22 ± 0.61 and 0.20 ± 0.03 GPa for the wet samples, respectively. Thus, compared with the wet samples, the elastic modulus and hardness of the dry samples increased by 38.32% and 40.01%, respectively. These results confirmed that water has a great influence on the elastic modulus and hardness of bony plates.

**Figure 7:**
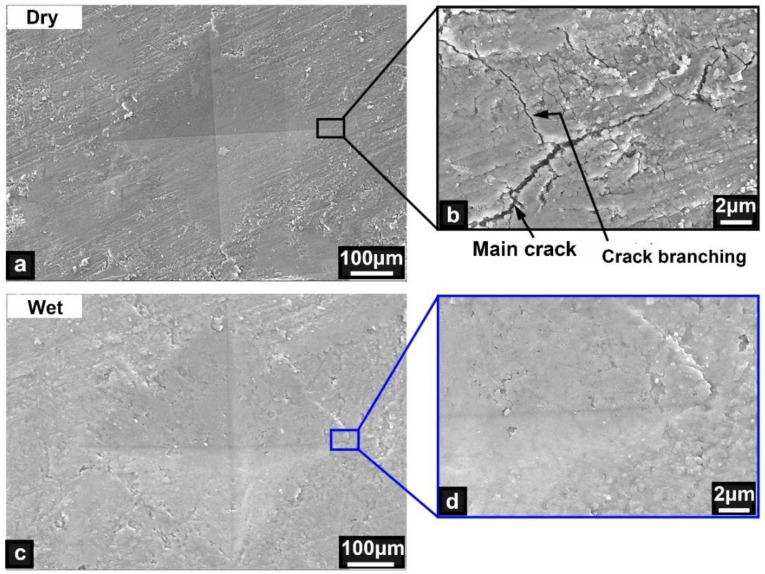
Microstructural features of the ventral bony plates after the nanoindentation tests. (**a**,**b**) Indentation and crack characteristics of the dry samples. (**c**,**d**) Indentation and local characteristics of the wet samples.

The microstructural characteristics of the nanoindentation samples were further examined, as shown in Figure 7. Cracks appeared at the end of the indentation for the dry samples (Figure 7a,b), whereas no such cracks were observed for the wet samples (Figure 7c,d). The cracking characteristics of the dry bony plates were consistent with those of brittle structural materials, that is, crack branches were generated during propagation of the main crack [31,32]. Therefore, the dry samples had obvious brittle characteristics. By contrast, the end of the indentation was smooth and flat for the wet samples, which revealed that water increased the toughness and elastic strain of the bony plates and effectively prevented the generation of cracks.

### 3.5. Tensile Responses

**Figure 8:**
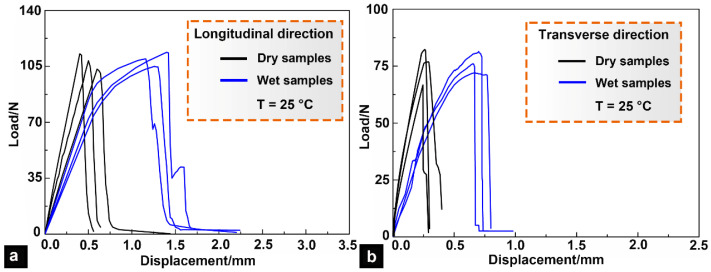
Tensile response curves for wet and dry bony plates. (**a**) Tensile response curves for the samples tested along the longitudinal direction. (**b**) Tensile response curves for the samples tested along the transverse direction.

The tensile mechanical responses of the dry and wet specimens in the longitudinal and transverse directions are plotted in Figure 8. The results demonstrate that the water content exerted little influence on the tensile strength of the bony plates. The response curves of the dry samples were linear, which implies that the dry bony plates displayed obvious brittle characteristics. By contrast, the response curves of the wet samples were bilinear, which indicates that elastic–plastic deformation occurred in the wet bony plates. Although the tensile strengths of the dry and wet samples in the same direction displayed little difference, the presence of water significantly improved the viscoelasticity and toughness of the mineral crystals and collagen fibers. Thus, the presence of water in the bony plates made the fibers easier to slide, deform, and rotate, thus greatly improving the ductility of the bony plates and effectively delaying their fracture failure.

Table 3 summarizes the tensile strengths of the samples. It can be seen that both the dry and wet specimens exhibited significant anisotropy, and the tensile strength in the longitudinal direction was approximately 39% higher than that in the transverse direction. Although the bony plates were very light, their specific tensile strength was nearly the same order as that of engineering alloys such as stainless steel (65 kN·m/kg) [33], which indicates that the bony plates were both lightweight and high strength.

### 3.6. Compression Properties

**Figure 9:**
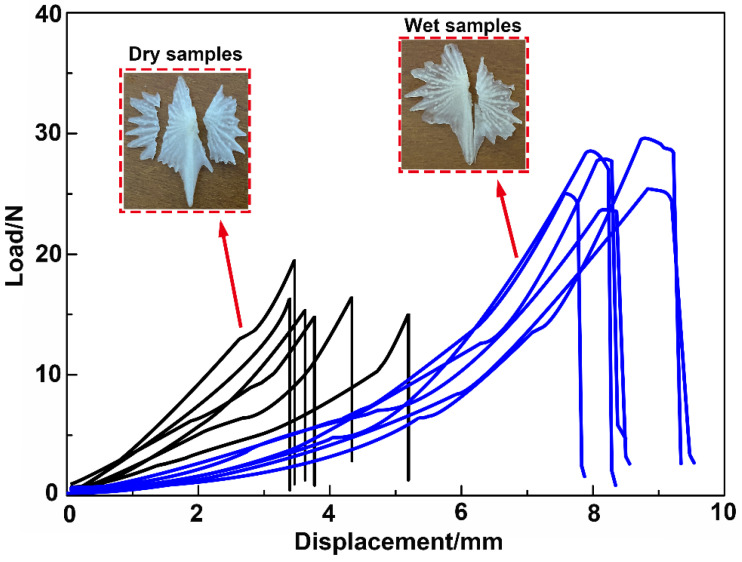
Compression tests for the wet and dry bony plates.

The compression force–displacement curves for the dry and wet samples are shown in Figure 9. The results revealed that the compression force for the wet samples was clearly higher than that for the dry samples. Compared with the dry samples, the maximum load and maximum displacement of the wet samples increased by 64.71% and 98.85%, respectively, which indicated that water significantly improved the elastic strain, toughness, and compression properties of the bony plates. The nanoindentation test results demonstrated that the elastic modulus and hardness of the dry samples were significantly higher than those of the wet samples, which led to a higher deformation resistance for the dry samples in the compression tests. It can also be seen from Figure 9 that the compression force of the dry samples was significantly higher than that of the wet samples under the same displacement. However, owing to the obvious brittleness of the dry samples, the bony plates on both sides of the crest broke prematurely during the compression tests (Figure 9). The wet samples gave full play to the advantages of the ridge-like structure: in the compression tests, the failure mode of the samples involved fracture of the crest structure (Figure 9). Because the crest structure had a greater thickness compared with the bony plates on either side, the fracture compression forces for the wet samples were greater than those for the dry samples.

Furthermore, the compression curve characteristics for the wet samples were distinct from those for the dry samples. The fracture behavior of the dry samples ended in an instant, such that the curves dropped sharply after reaching the maximum load. By contrast, the fracture behavior of the wet samples could be approximately regarded as a gradual process: the fracture of the crest structure occurred gradually from the surface layer to the internal layer, from the anterior side to the posterior side of the crest, and this behavior caused the compression curves to produce small fluctuations after reaching the maximum load. The curves then dropped sharply until the fracture process had ended. These results demonstrate that, compared with the dry samples, the wet bony plates could give full play to the deformation characteristics endowed by their structure and shape and thereby afford the best protective properties.

## 4. Discussion

In this work, we studied the morphological characteristics and protective mechanisms of the ventral bony plates in the Chinese sturgeon, and the results indicated that the bony plates had unique protective functions. The bony plates possessed a multilayer structure composed of a mineralized crystal surface layer and a mineralized collagen fiber layer. The mineralization degree of the surface layer was higher than that of the internal layer, which provided excellent strength for the bony plates; the mineralized fibrous tissues in the internal layer could effectively improve the fracture toughness and ductility of the bony plates. Moreover, compared with typical fish scales, the ridge-like bony plates displayed stronger deformation ability to effectively resist the compression load. In addition, the bony plates possessed sharp serrated edges, which may effectively damage attacking predators [34].

According to the results of the nanoindentation, tension, and compression tests, the excellent mechanical protective properties of the bony plates can be summarized into the following three aspects: (1) the highly mineralized tissues effectively improved the elastic modulus and hardness of the bony plates, thereby enhancing their strength; (2) water significantly reduced the brittleness of the bony plates and effectively delayed crack initiation and propagation, thus avoiding premature fracture failure of the structures; and (3) the ridge-like shape endowed the bony plates with great deformation capacity, such that they could absorb a higher load through deformation.

Water exerted a profound influence on the nanoindentation and compression properties of the bony plates, which can be explained from the interaction mechanisms between water, collagen fibers, and mineralized materials [35,36]. With increasing water content, the collagen matrix and water molecules increase the viscoelasticity of the tissues, which allows the collagen fibers to more easily slide, rotate, and deform, thereby increasing the elastic strain, toughness, and deformation capacity of the bony plates. Meanwhile, the bony layer of the dry samples would shrink, and the collagen fibers would become stiffer, thus increasing the elastic modulus and hardness of the bony plates. Although the dry samples exhibited higher strength and hardness, the dry bony plates were likely to fracture rapidly when cracks occurred owing to their obvious brittle characteristics, such that these samples could not give full play to the deformation and energy absorption properties of the structure. Although a higher water content reduced the elastic modulus and hardness of the bony plates, it also greatly improved the toughness and elastic strain of the structures and enhanced the deformability and fracture toughness of the bony plates. Thus, the wet bony plates effectively balanced the strength, hardness, and toughness, thereby significantly delaying the fracture failure of the structures.

Furthermore, the ventral bony plates of the Chinese sturgeon displayed obvious anisotropic characteristics. Anisotropy is a remarkable feature of biological structures, which is caused by an uneven and discontinuous material distribution [12]. These biological structures often exhibit distinct mechanical properties in different directions to meet particular functional requirements. In the current experiments, the deformation direction of the ventral bony plates of the Chinese sturgeon was from the crest to the serrated edges (longitudinal direction, as shown in Figure 1c), and the collagen fibers in the internal layer of the bony plates were also arranged along the longitudinal direction (Figure 4d). These features indicate that the tensile response demands of the bony plates in the longitudinal direction were significantly higher than those in the transverse direction.

## 5. Conclusions

In this work, we comprehensively studied the microstructural characteristics and mechanical properties of the ventral bony plates of the Chinese sturgeon and further investigated the damage mechanisms of the bony plates. The results revealed that the bony plates had excellent protective performance. The following conclusions can be drawn:

(1) The ventral bony plates possessed a ridge-like structure with serrated edges. There were numerous nodular ridges on the surface of the bony plates with a height of 200–300 μm. These nodular ridges enhanced the connectivity between the bony plates and the fish skin epidermis. The bony plates featured a hierarchical structure with a thickness of approximately 300 μm that was composed of highly mineralized nanocrystals and highly mineralized collagen fibers.
(2) The bony plates were primarily composed of HAP. Along the surface layer to the internal layer of the bony plates, the Ca, P, and O contents gradually decreased, while the C content increased. This indicates that the mineralization degree of the internal layer decreased while the content of organic tissues such as mineralized collagen fibers increased. The composition analysis results were consistent with the microscopic morphological observations. The presence of fibrous tissues significantly improved the fracture mechanical properties of the bony plates.
(3) The results of nanoindentation tests revealed that the elastic modulus and hardness of the bony plates decreased gradually from the surface layer to the internal layer. Compared with the wet samples, the elastic modulus and hardness of the dry samples were 38.32% and 40.01% higher, respectively. It is readily apparent that the presence of water significantly reduced the elastic modulus and hardness of the bony plates. However, the indentation microstructure also indicated that water effectively improved the elastic strain and toughness of the bony plates and delayed crack initiation and propagation, thereby greatly enhancing the fracture resistance of the structures.
(4) The results of tension tests demonstrated that the bony plates had obvious anisotropic characteristics. The tensile strengths of the dry and wet specimens were 136.23 ± 3.57 and 139.47 ± 4.26 MPa in the longitudinal direction and 98.62 ± 3.43 and 100.57 ± 3.51 Mpa in the transverse direction, respectively. For the dry and wet samples, the tensile strengths in the longitudinal direction were 38.14% and 38.68% higher, respectively, than those in the transverse direction. In addition, water had no effect on the tensile strength of the bony plates, although it effectively improved the viscoelasticity and ductility of the structures and enabled easier sliding and rotation of the collagen fibers, thus improving the plasticity of the bony plates.
(5) The results of compression tests revealed that the compression force for the wet samples was increased by 64.71% compared with the dry samples. Although the dry samples displayed superior elastic modulus, hardness, and deformation resistance, fracture failure occurred prematurely during the tests owing to the obvious brittleness of these samples. By contrast, the wet specimens exhibited good elastic strain and toughness and could fully exploit the deformation characteristics of the bony plates. Thus, the wet bony plates afforded superior protective performance by effectively balancing strength, hardness, and toughness, which could provide unique guidance and insights for the design of lightweight protective structures.

## Figures and Tables

**Table 1:** Elemental contents in the structural layers of the ventral bony plates.

Element	Surface Layer	Internal Layer
C	25.32	35.59
Ca	31.55	25.41
P	19.58	16.52
O	18.75	16.63

**Table 2:** Average elastic modulus and hardness values for the ventral bony plates.

Sample	Layer	Modulus/GPa	Hardness/GPa
Dry	Surface layer	11.85 ± 0.63	0.29 ± 0.05
	Internal layer	10.88 ± 0.78	0.26 ± 0.04
Wet	Surface layer	8.69 ± 0.67	0.22 ± 0.04
	Internal layer	7.74 ± 0.58	0.18 ± 0.03

**Table 3:** Tensile strengths of the ventral bony plates.

	Dry Samples	Wet Samples
Longitudinal tensile strength (MPa)	136.23 ± 3.57	139.47 ± 4.26
Transverse tensile strength (MPa)	98.62 ± 3.43	100.57 ± 3.51
Longitudinal specific tensile strength (kN·m/kg)	46.19 ± 2.14	48.09 ± 2.27
Transverse specific tensile strength (kN·m/kg)	32.26 ± 2.22	33.63 ± 2.18

## Data Availability

Data are only available upon request due to restrictions regarding, e.g., privacy and ethics. The data presented in this study are available from the corresponding author upon request. The data are not publicly available due to their relation to other ongoing research.
